# Identification of DNA N6-methyladenine modifications in the rice genome with a fine-tuned large language model

**DOI:** 10.3389/fpls.2025.1626539

**Published:** 2025-06-25

**Authors:** Yichi Zhang, Hao Chen, Shicheng Xiang, Zhibin Lv

**Affiliations:** College of Biomedical Engineering, Sichuan University, Chengdu, China

**Keywords:** rice genome, N6-methyladenine, large language model, BERT, UMAP visualization

## Abstract

DNA N6-methyladenine (6mA) plays a significant role in various biological processes. In the rice genome, 6mA is involved in important processes such as growth and development, influencing gene expression. Therefore, identifying the 6mA locus in rice is crucial for understanding its complex gene expression regulatory system. Although several useful prediction models have been proposed, there is still room for improvement. To address this, we propose an architecture named iRice6mA-LMXGB that integrates a fine-tuned large language model to identify the 6mA locus in rice. Specifically, our method consists of two main components: (1) a BERT model for feature extraction and (2) an XGBoost module for 6mA classification. We utilize a pre-trained DNABERT-2 model to initialize the parameters of the BERT component. Through transfer learning, we fine-tune the model on the rice 6mA recognition task, converting raw DNA sequences into high-dimensional feature vectors. These features are then processed by an XGBoost algorithm to generate predictions. To further validate the effectiveness of our fine-tuning strategy, we employ UMAP(Uniform Manifold Approximation and Projection) visualization. Our approach achieves a validation accuracy of 0.9903 in a five-fold cross-validation setting and produces a receiver operating characteristic (ROC) curve with an area under the curve (AUC) of 0.9994. Compared to existing predictors trained on the same dataset, our method demonstrates superior performance. This study provides a powerful tool for advancing research in rice 6mA epigenetics.

## 1 Introduction

N6-methyladenine(6mA) is produced by methylation of the N6 position of adenine and has been found in bacteria, eukaryotes, and archaea (Zhang et al., 2015; O’Brown and Greer, 2016). Rice is one of the most important cereal crops in the world. Within the rice genome, 6mA serves as a critical epigenetic modification, regulating gene expression through methylation at the N6 position of adenine (Lv et al., 2020; Chen et al., 2022; Jin et al., 2022). Studies have shown that 6mA in rice plays a vital role in many biological functions. For example, 6mA in rice is associated with stress response and helps rice to better adapt to adversity (Zhang et al., 2018; Ding et al., 2023). It is also associated with reproduction and regulates the growth and development of rice (Zhou et al., 2021; Yang et al., 2024). Zhou et al. discovered that 6mA is highly enriched in specific sequence motifs, conserved DNA sequence patterns that serve as recognition sites for epigenetic regulators. These motifs include AGG and GAGG, which are assumed to represent the binding elements of methyltransferase complexes or chromatin associated proteins. 6mA methylation preferentially occurred on these specific nucleotide motifs, indicating their functional significance in epigenetic regulation (Lee et al., 2018). And this methylation pattern is tightly linked to the drought stress response in rice (Zhou et al., 2018; Yang et al., 2024). In addition, 6mA can directly affect seed size and yield formation by regulating the expression of endosperm development-related genes (Zhou et al., 2021). In recent years, epigenetic breeding strategies based on CRISPR-6mA editing technology have provided new ideas to improve disease resistance and yield in rice by targeting modification of the 6mA locus (Romero and Gatica-Arias, 2019). However, traditional experimental methods such as SMRT-seq for detecting 6mA locus have the limitations of high cost and low throughput, and there is an urgent need to develop efficient computational prediction models to guide subsequent functional studies (Zhu et al., 2018; Wang L. et al., 2023; Chen et al., 2024; Liu et al., 2024; Shao et al., 2024; Xie H. et al., 2024; Zhou et al., 2024).

In recent years, machine and deep learning approaches have successfully addressed many challenges in identifying 6mA modifications in rice genomes (Sinha et al., 2023; Wang R. et al., 2023). In 2019, Chen et al. developed the first method for predicting DNA 6mA sites in rice, called i6mA-Pred, utilizing nucleotide chemical property (NCP) features and a support vector machine (SVM) as the classifier (Chen et al., 2019; Zou et al., 2022; Meher et al., 2024; Wang Y. et al., 2024). Subsequent research has seen the emergence of various single-classifier-based prediction methods, including MM-6mAPred (Pian et al., 2019), i6mA-DNCP (Park et al., 2020), iN6-methylat (Le, 2019), and iDNA6mA-rice (Lv et al., 2019). Moreover, ensemble learning models combining multiple classifiers, such as csDMA (Liu et al., 2019), SDM6A (Basith et al., 2019), 6mA-Finder (Xu et al., 2020), Meta-i6mA (Hasan et al., 2021), i6mA-VC (Xue et al., 2021), i6mA-Vote (Teng et al., 2022), and EpiSemble (Sinha et al., 2023), have been developed to enhance model performance and robustness. Deep learning techniques have evolved from traditional artificial neural network frameworks and have shown significant improvement in predictive power across multiple research domains. With the development of deep learning and its excellent performance, researchers began to apply it to the problem of DNA 6mA site prediction. In 2019, Yu et al. developed a prediction model called SNNRice6mA (Yu and Dai, 2019) based on convolutional neural networks (CNNs) through single-nucleotide one-hot coding, obtaining an accuracy of 0.920. Another group of researchers, Lv et al., proposed a convolutional neural network iRicem6A-CNN (Lv et al., 2021) based on a dinucleotide one-hot encoder in 2020, achieving an accuracy of 0.938 for 5-fold cross-validation. However, it is worth noting that CNNs are limited in focusing on only part of the information. Deep6mA (Li Z. et al., 2021), which consists of a convolutional neural network (CNN) and a bidirectional LSTM (BLSTM) module to solve the long-distance nucleotide association problem by learning contextual dependencies of the sequences, was proposed by Li et al. in 2021 and achieved a 5-fold cross-validation accuracy of 0.940.

Over the last few years, large-scale language modeling (LLM) has progressed tremendously (Li H. et al., 2021; Xie X. et al., 2024; Chen et al., 2025). The well-known model, ChatGPT, is a fine-tuned version of the base GPT-3 model. By learning contextual text in a self-supervised manner, it can both understand and generate human language (Devlin et al., 2019; Wang G. et al., 2024). DNA sequences exhibit similarities to natural language. Nucleotides, the building blocks of nucleic acids, serve as “words” within biological systems’ “languages”. LLMs can be adapted for the analysis of biological sequence data by leveraging the structure of DNA and protein sequences as analogous to natural language texts (Jumper et al., 2021; Rives et al., 2021; Wei et al., 2021; Li T. et al., 2024; Li Y. et al., 2024; Qiao et al., 2024; Lai et al., 2025; Xie et al., 2025). There have been many breakthroughs in LLMs for applications in biology, such as AlphaFold2, a protein prediction model with very high accuracy (Jumper et al., 2021), the Geneformer model trained on data from about 10 million human single-cell RNA sequences (Zou et al., 2019; Theodoris et al., 2023), and DNABERT, a transformer-based DNA pre-training model (Ji et al., 2021). While LLMs demonstrate potential for identifying patterns and correlations in noisy biological datasets (Lam et al., 2024; Soylu and Sefer, 2024; Xie X. et al., 2024; Liu et al., 2025), they have yet to gain acceptance within plant science research. To date, LLMs have not been employed in the study of 6mA locus prediction in rice.

In this study, we develop a large language model-based transfer learning model called iRice6mA-LMXGB. it consists of a pre-trained DNABERT2 model and an XGBoost model. It contains a unique fine-tuning architecture that relies exclusively on DNA sequence data to distinguish 6mA sequences from non-6mA sequences. Experimental results demonstrate the model’s outstanding performance, achieving a validation accuracy of 0.9903 through 5-fold cross-validation. Compared to all previous methods tested on standard datasets, iRice6mA-LMXGB significantly outperforms them, suggesting that this novel approach has the potential to transform biological sequence modeling.

## 2 Materials and methods

### 2.1 Benchmark dataset

In this study, we utilized the rice dataset constructed by Lv et al. (2020) for model training and evaluation using 5-fold cross-validation. To ensure the high quality of the data, sequences with greater than 80% similarity were removed via the CD-HIT program (Li and Godzik, 2006). The dataset is made of 154,000 sequences with 6mA sites and 154,000 sequences without 6mA sites. This is a widely adopted and balanced rice dataset. During model training, unbalanced datasets may lead to unreliable results. The majority class samples are dominant and the model will favor the majority class during training, thus ignoring the minority class. This may result in the model having high accuracy for the majority class but low recognition for the minority class during prediction. For ease of reference, we denote it as “rice-Lv” throughout this study. Both positive and negative sequences in the rice-Lv dataset are 41 base pairs in length. Positive sequences represent 6mA modifications at their centers, while negative sequences lack such modifications at theirs. By employing this well-established dataset, we enable a fair comparison between our method and those previously reported.

### 2.2 Architecture of iRice6mA-LMXGB

**Figure 1:**
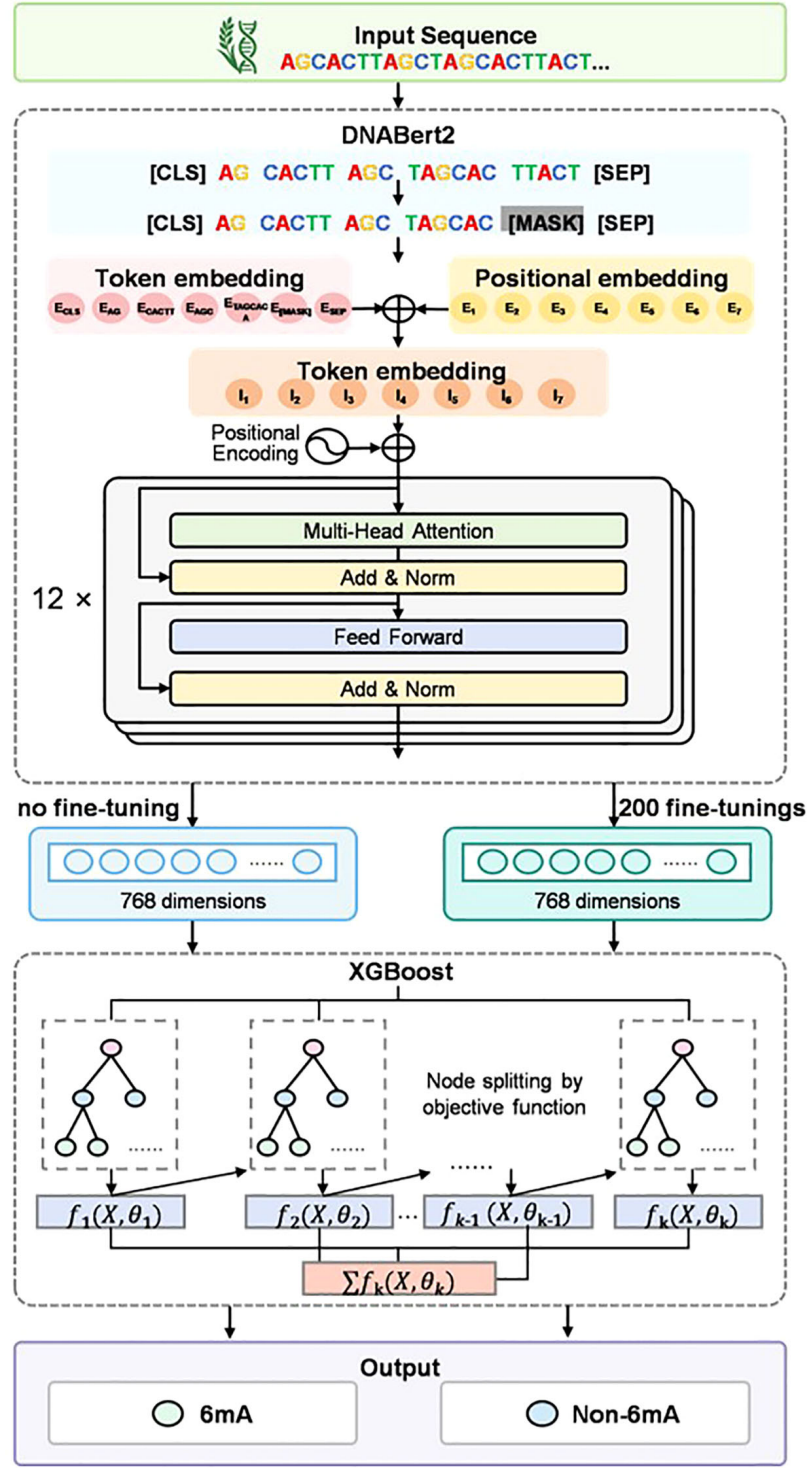
The proposed modeling framework, iRice6mA-LMXGB.

The architecture of iRice6A-LMXGB is presented in **Figure 1**, comprising two main components: the pre-trained DNABERT-2 module and the XGBoost module. DNABERT-2 is a pre-trained BERT model specifically designed for encoding DNA sequences. It can efficiently identify complex long-range dependencies in these sequences (Zhou et al., 2023). And this module will undergo further fine-tuning in this study. XGBoost’s superior performance, particularly in terms of speed and accuracy when processing large-scale datasets, enables its extensive use in solving classification problems (Chen and Guestrin, 2016; Yang et al., 2021). It utilizes the feature vectors output from the DNABERT-2 model to generate final prediction results. A detailed explanation of the model follows.

#### 2.2.1 DNABERT-2

DNABERT-2 is an iterative version of DNABERT. DNABERT is the first BERT-based DNA language model (Ji et al., 2021). Rigorously trained on a comprehensive genomic dataset encompassing the entire human genome, DNABERT offers a linguistic perspective for genomic analysis. While widely adopted, the initial version of DNABERT exhibited notable technical limitations. Specifically, DNABERT faced two critical challenges: first, its training data is limited to a single-species genome, which makes it difficult for the model to capture sequence-conserving patterns and diversity features across species; second, the k-mer sequence partitioning mechanism it employs not only triggers the hidden danger of data leakage during the training process, but also significantly increases the computational complexity (Moeckel et al., 2024). Such limitations underscore the pressing need for innovation and improvement in DNA-based language modeling research. To address these challenges, DNABERT-2 introduced significant improvements in both areas. First, it breaks through species boundaries and employs cross-species genomic datasets for pre-training, significantly enhancing the model’s ability to recognize evolutionarily conserved regions and species specificity. Second, at the data processing stage, DNABERT-2 employs byte-pair encoding (BPE), a novel tokenization method that replaces traditional k-mer partitioning. This is a data compression algorithm widely used in large-scale language models (Sennrich et al., 2015), which effectively solves the risk of data leakage and improves computational efficiency, successfully overcoming the limitations of k-mer tokenization. As demonstrated by Zhihan et al.’s comparative analysis, compared to conventional 6-mer tokenization methods, the byte-pair encoding (BPE) implementation exhibits superior sequence compression efficiency, reducing the tokenized sequence length by a factor of 5. The dramatic reduction in dimensionality directly improves the computational efficiency of processing genome sequences (Zhou et al., 2023).

The BERT model consists of two independent components: the module responsible for preprocessing BERT input and the pre-training BERT module. In the BERT input preprocessing module, DNABERT-2 utilizes BPE to tokenize DNA sequences. Byte Pair Encoding (BPE) is a subword tokenization algorithm commonly employed in NLP Natural Language Processing) tasks. Its key mechanism lies in iteratively merging character pairs of the highest frequency to construct a vocabulary of subwords. During tokenization, DNABERT-2 appends a [CLS] token at the sequence start and a [SEP] token at the end. Then, each token is put into an embedding module and converted into a vector. The DNABERT-2 model uses the ALiBi(Attention with Linear Biases) (Press et al., 2021) approach, which does not add positional embeddings to the input, but rather adds a non-learned embedding in every Attention computation to add a non-learning bias and a fixed set of statics to combine the location information with the Attention score. DNABERT-2 employs a transformer encoder architecture as the backbone of its pre-trained BERT module. The feature matrix is constructed by cascading encoders layer by layer across the network’s layers (L). Each encoder comprises three components: multi-head self-attention units, position-wise feed-forward neural networks, and normalization layers. Within the i-th encoder stage, the multi-head self-attention mechanism operates as follows.


$$\text{Multihead}(X^{i})=\text{Concat}(head_{1},head_{2},\ldots ,head_{n})W^{O,i}$$

For the i-th encoder, the input matrix $X^{i}\;$ is handled through n self-attentive heads for processing. The outputs of these heads are then transformed by the output transformation matrix $W^{o,i}$ , which is computed in detail for each $head^{i}$ as follows.


$${\text{head}}^{i}=\text{softmax}(\frac{W^{Q,i}X^{i}{\left(W^{K,i}X^{i}\right)}^{T}}{\sqrt{d_{k}}})\;W^{V,i}X^{i}$$

$W^{Q,i}$ , $W^{K,i}$ and $W^{V,i}$ serve as the transformation matrices for the query, key, and value components of each head, respectively. $d_{k}$ denotes the dimension of the matrix.

Specifically, after computing $MultiHead(X^{i})$ in the multi-head attention mechanism, this resultant output is added to the residual connection of the original input $X^{i}$ for normalization. The computation proceeds according to the formula below.


$$Y^{i}=\text{LayerNorm}(\text{MultiHead}(X^{i})+X^{i})$$

After normalization, the processed data is passed through a feed-forward neural network using the following formula:


$$FFN(Y^{i})=\max(0,Y^{i}W_{1}+b_{1})W_{2}+b_{2}$$

$W_{1}$ , $W_{2}$ , $b_{1}$ and $b_{2}$ are the trainable weight parameters within the feed-forward layer.

The output of the i-th encoder is achieved through normalization of the residual connection between $Y^{i}$ and $FFN(Y^{i})$ . Below is the corresponding formula.


$$X^{i+1}=\text{LayerNorm}(Y^{i}+\text{FFN}(Y^{i}))$$

Finally, the output of the DNABERT-2 can be obtained by cascading the L encoders as follows.


$$X_{1}=X^{i+1}\in R^{d\times N}$$

where d denotes the dimension of the word vector and N represents the total number of tokens.

DNABERT-2 follows the BERT model architecture, defined by three key parameters: L = 12, H = 768, and A = 12. The parameter L specifies the number of transformer layers (totaling 12). The parameter H determines the hidden layer size, with each token represented as a 768-dimensional vector. The parameter A specifies the number of attention heads (totaling 12). In this study, we use the full fine-tuning (FFT) (Church et al., 2021) method, which treats rice DNA sequences as “sentences in natural language” and inputs them into the DNABERT-2 module to adjust and update all the parameters. Finally, we use the BERT model to convert them into fixed-length feature vectors to obtain the original feature matrix before fine-tuning and the feature matrix after 200 cycles of updating.

#### 2.2.2 XGBOOST

The XGBoost classifier is a gradient boosting method that integrates regression trees (Basith et al., 2019). The objective function of the model is obj(θ)=L(θ)+Ω(θ) , L(θ) is the training loss function with the expression:


$$L(\text{θ})=\sum_{i=1}^{n}l(y_{i},\hat{y_{i}})$$

Where $l(y_{i},\hat{y_{i}})$ represents the training loss function for each sample. $y_{i}$ represents the true value of the i-th sample. $\hat{y_{i}}$ represents the estimated value of the i-th sample.

Then the estimated value of the i-th sample is expressed as:


$$\hat{y_{i}}=\sum_{k=1}^{K}f_{k}(x_{i}),f_{k}\in F$$

K is the number of integrated trees, and F denotes the space of all possible decision trees. $f_{k}$ is a specific categorical regression tree (CART). Ω(f) is the tree structure complexity function, and its specific form is:


$$\text{Ω}(f)=\text{γ}T+\frac{1}{2}\text{λ}\sum_{i=1}^{T}w_{i}^{2}$$

The parameter γ restricts the number of leaf nodes T of the tree to control the complexity of the model. And the parameter λ constrains the sum of the weights $w_{i}^{2}\;$ of each leaf node to suppress overfitting. The objective function is continuously optimized by adjusting the parameters for the optimal result. In this way, the XGBoost classifier finally outputs the prediction results of the rice sequence about 6mA by receiving the extracted feature vectors from DNABERT-2.

### 2.3 Evaluation metrics and methods

In this study, we validate our approach using a traditional 5-fold cross-validation method and compare it to previous studies based on the benchmark dataset rice-Lv.

we will combine five metrics, including accuracy (ACC), sensitivity (Sn), specificity (Sp), Matthew’s correlation coefficient (MCC), and area under the curve (AUC), to comprehensively evaluate the prediction performance of our model (Zou et al., 2023; Zulfiqar et al., 2023; Guo et al., 2024; Huang et al., 2024; Zhu et al., 2024).

ACC indicates the overall correctness of the model prediction and is a basic benchmark used to evaluate the model performance, which can be expressed as:


$$ACC=\frac{TP+TN}{TP+TN+FP+FN}$$

The sensitivity Sn, also known as the true positive rate (TPR), is expressed as:


$$SN=\frac{TP}{TP+FN}$$

The specificity Sp, also known as the true negative rate (TNR), is expressed as:


$$SP=\frac{TN}{TN+FP}$$

MCC is a composite metric that assesses the overall quality of classification model predictions by examining the performance of the classification model in each of the four quadrants of the confusion matrix. The superior score reflects the balanced excellence between true positives (TP), true negatives (TN), false negatives (FN) and false positives (FP). It can be defined as:


$$MCC=\frac{TP\times TN-FP\times FN}{\sqrt{\left(TP+FP)(TP+FN)(TN+FP)(TN+FN\right)}}$$

The last performance metric we use is AUC, defined as the value of the area under the subject’s operating characteristic curve. AUC is also an important measure of the performance of a dichotomous model. The larger the value of AUC, the better the model performs. AUC is a floating-point number between 0 and 1. 1 indicates that the model predicts perfectly, whereas 0.5 indicates that the model is similar to a random prediction (Zhang et al., 2025).

## 3 Results and discussion

### 3.1 Model performance analysis

**Figure 2:**
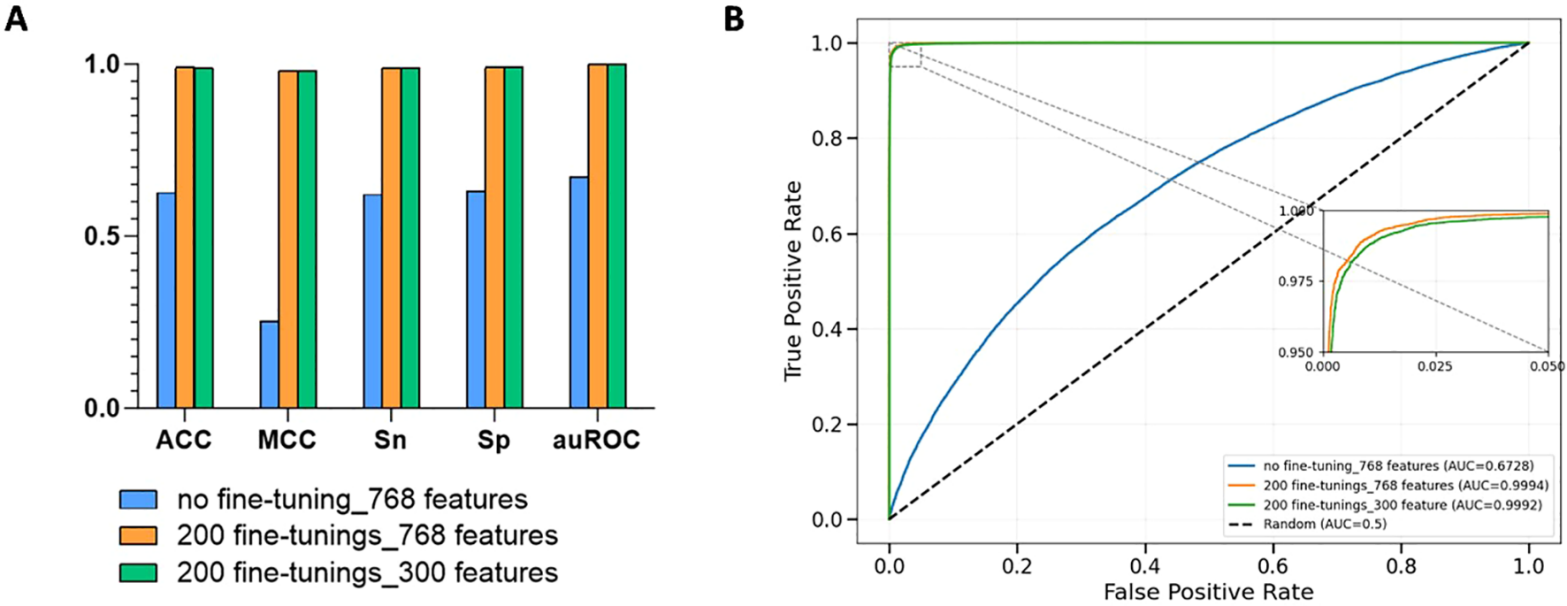
**(A)** Comparison of model performance with or without fine-tuning and with or without feature selection; **(B)** Average ROC curves for five-fold cross-validation of the three models. Where no fine-tuning_768 features denotes the model with no fine-tuning, 200 fine-tunings_768 features denotes the model with two hundred fine-tunings without feature selection, and 200 fine-tunings_300 features denotes the model that was fine-tuned 200 times and ranked for feature importance and the top 300 features are selected after the feature importance ranking.

In this study, we developed three models. For the first model, we directly used the pre-trained DNABERT-2 to extract 768-dimensional features from rice DNA and fed them into an XGBoost classifier for prediction tasks. The XGBoost classifier shows unique advantages in genomics data classification tasks, mainly due to its ability to efficiently handle high-dimensional sparse data and its built-in regularization mechanism. Our dataset, with more than 300,000 samples, is characterized by high feature dimensionality, and XGBoost is able to efficiently capture nonlinear interaction effects through the gradient boosting framework combined with second-order derivative optimization. Its regularization term can in turn suppress overfitting and enhance model generalization (Chen and Guestrin, 2016). Cross-validation results showed ACC=0.6259, Sn=0.6207, Sp=0.6312, MCC=0.2519, and auROC=0.6728 for this configuration. For the second model, we loaded the rice-Lv dataset into the DNABERT-2 module and conducted 200 iteration loops to develop a fine-tuned version of the model. The 5-fold cross-validation scores were ACC=0.9903, Sn=0.9898, Sp=0.9907, MCC=0.9805, auROC=0.9994 which are 58.22%, 59.47%, 56.96%, 289.24%, and 48.54%, respectively higher than those of the non-fine-tuned model. For the third model, we utilized LightGBM’s built-in function to assess and prioritize feature importance using features extracted from the fine-tuned DNABERT-2 model (Ke et al., 2017). The feature ranking principle of LightGBM is based on the Gradient Boosting Decision Tree (GBDT) framework, which evaluates feature importance by quantifying the contribution of features in the process of constructing the decision tree (Ke et al., 2017). Following this, we selected the top 300 features for modeling with XGBoost. The 5-fold cross-validation yielded ACC=0.9899, Sn=0.9890, Sp=0.9908, MCC=0.9799, and auROC=0.9994. As shown in **Figure 2**, our cross-validation results indicate that: (1) Fine-tuned models outperformed non-fine-tuned counterparts significantly. (2) However, applying feature selection after fine-tuning caused minor performance degradation compared to models without feature selection, not much difference overall. These findings demonstrate the effectiveness of our fine-tuning strategy. The pre-training model is usually trained on multi species datasets, and may not be able to capture the 6mA distribution pattern unique to rice. Through the fine-tuning strategy, the model parameters are recalibrated, which can give priority to the local features in the rice genome, and the sensitivity of the model to the sequence context of rice 6mA is improved. Additionally, while XGBoost’s tree-based architecture excels at managing high-dimensional data through regularization techniques, our results suggest that applying LightGBM-based feature selection after fine-tuning may slightly reduce model performance due to fewer feature interactions. We selected the second model with the best performance, performing fine-tuning for 200 iterations without feature selection, to name iRice6mA-LMXGB.

### 3.2 UMAP dimensionality reduction visualization

In order to perform an in-depth analysis of the interpretability of the iRice6mA-LMXGB model after integrating DNABERT-2 with XGBoost, we used the UMAP (Uniform Manifold Approximation and Projection) technique. This is a nonlinear dimensionality reduction and visualization algorithm for large-scale datasets. Umap assumes that the data is distributed on a low dimensional manifold. Firstly, the probability weight is defined in the high dimensional space using the neighborhood graph to reflect the similarity between points. Then the cross entropy loss function is used to optimize the embedding in the low dimensional space to align the low dimensional similarity with the high dimensional structure. Based on graph theory and flow learning methods, it is assumed that the available data samples are uniformly distributed in the topological space and can be approximated and mapped from these finite data samples to a lower-dimensional space for visualization and analysis (McInnes and Healy, 2018).

**Figure 3:**
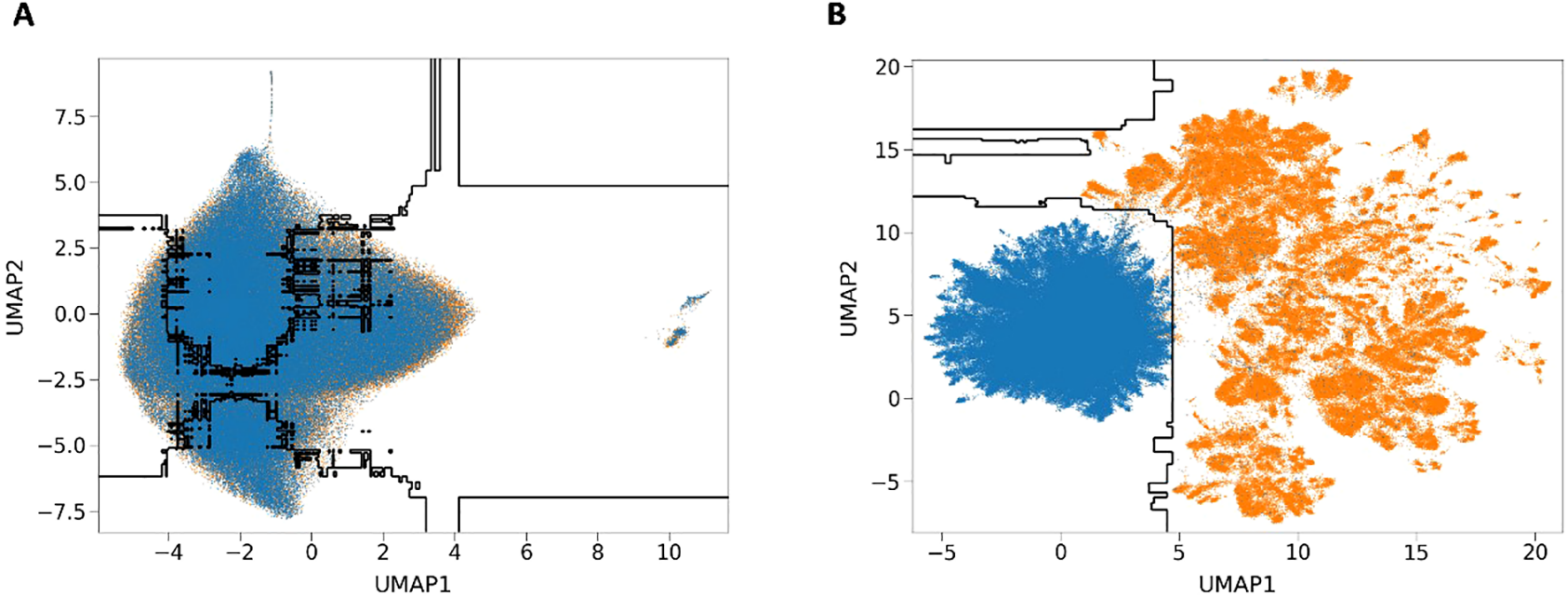
UMAP dimensionality reduction visualization. **(A)** UMAP results of the original features of the unfine-tuned model. **(B)** UMAP results of features learned by the iRice6mA-LMXGB model.

To be more specific, we will visualize the distribution of 6mA and non-6mA by projecting each feature vector onto a 2D view using the UMAP technique. **Figure 3** shows the arrangement of 6mA and non-6mA samples in 2D space before and after fine-tuning, and the decision boundary drawn in black by the XGBoost algorithm. Blue markers denote non-6mA samples, and orange markers denote 6mA samples. The first subplot represents the UMAP results of the original features without fine-tuning, which can be interpreted as all the sample points not showing any representative clustering. In **Figure 3A**, poor separation indicates significant feature overlap between the 6mA sample points and the non-6mA sample points (**Figure 3A**), suggesting a high degree of overlap in their distributions. The second subfigure shows the results of projecting the high-dimensional feature space learned from the iRice6mA-LMXGB model into a 2D view, which shows much improved clustering, indicating a significant increase in separation and a decrease in overlap in the feature space (**Figure 3B**), resulting in improved performance. In summary, our approach allows for better learning of model decision boundaries. Through this visualization technique, we can more intuitively understand the impact of features on model predictions, further deepening our exploration of model interpretability.

### 3.3 Comparison of the proposed model with existing models

**Figure 4:**
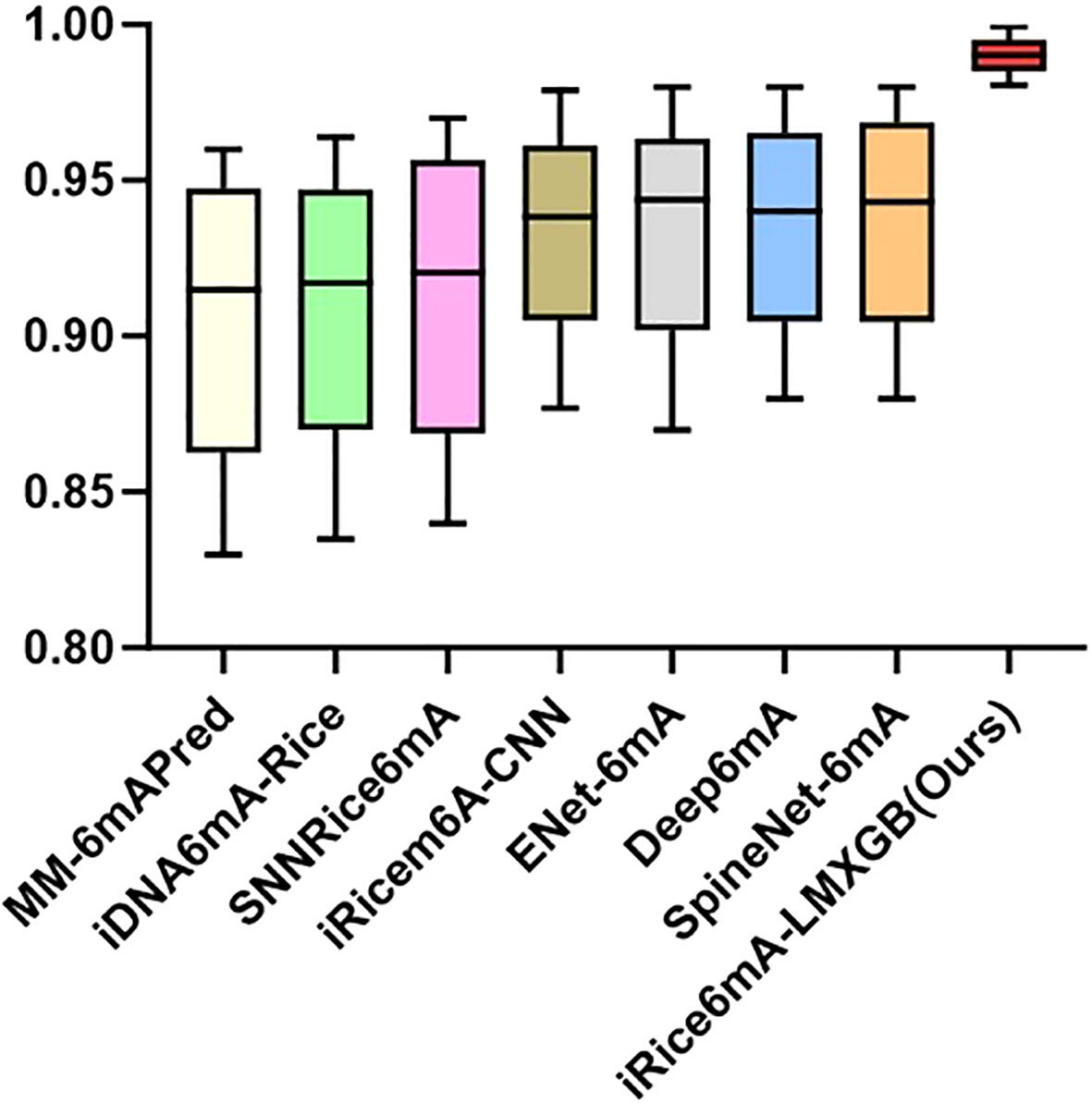
Comparison of the proposed model with other existing models on the rice-Lv dataset.

To better evaluate the performance of our model, we compare it with the following state-of-the-art methods, including MM-6mAPred (Pian et al., 2019), iDNA6mA-Rice (Lv et al., 2019), SNNRice6mA (Yu and Dai, 2019), iRicem6A-CNN (Lv et al., 2021), ENet-6mA (Abbas et al., 2022), Deep6mA (Li Z. et al., 2021) and SpineNet-6mA (Abbas et al., 2020). Our model is evaluated using the same five-fold cross-validation protocol on the same dataset as previous studies, employing the identical metrics: ACC, MCC, Sn, Sp, and AUC. As shown in **Table 1**, our iRice6mA-LMXGB model outperforms all previous predictors across all metrics and demonstrates more stable performance with less fluctuation in ACC, MCC, Sn, Sp, and AUC values. In ACC, MCC, Sn, and AUROC metrics, our model improves over the previous best predictor SpineNet-6mA by 5%, 11.42%, 3.42%, 6.62%, and 1.98%, respectively. Furthermore, it outperforms the previous best model, ENet-6mA, by 6.08% in Sp metric. To facilitate visualization of the comparison results, we created a box-and-whisker plot, as illustrated in **Figure 4**. To sum up, our iRice6mA-LMXGB model demonstrates superior performance compared to both machine learning-based and CNN/LSTM-based deep learning models for 6mA prediction in rice, showcasing its robustness as a predictive tool.

**Table 1:** 5-fold cross-validation results of iRice6mA-LMXGB with several previous methods on the rice-Lv dataset.

Method	ACC	MCC	Sn	Sp	AUROC
MM-6mAPred	0.9149	0.8300	0.9347	0.8951	0.9600
iDNA6mA-Rice	0.9170	0.8350	0.9300	0.9050	0.9640
SNNRice6mA	0.9204	0.8400	0.9433	0.8975	0.9700
iRicem6A-CNN	0.9382	0.8770	0.9434	0.9331	0.9790
ENet-6mA	0.9437	0.8700	0.9467	0.9339	0.9800
Deep6mA	0.9401	0.8800	0.9506	0.9296	0.9800
SpineNet-6mA	0.9431	0.8800	0.9571	0.9292	0.9800
iRice6mA-LMXGB (ours)	**0.9903**	**0.9805**	**0.9898**	**0.9907**	**0.9994**

Bold values indicate that the model proposed in this study achieves optimal results in each of the assessment metrics.

## 4 Conclusions

In this article, we develop a novel computational model called iRice6mA-LMXGB that combines fine-tuned large language modeling to efficiently distinguish and identify 6mA and non-6mA loci in the rice genome. We utilized the large language model, DNABERT-2, to represent the DNA sequence as a continuous word vector, thus effectively capturing the DNA sequence features. Subsequently, we applied the robust machine learning method XGBoost to make accurate predictions based on the extracted features. We compare and analyze the performance of iRice6mA-LMXGB with other predictors, and the results show that iRice6mA-LMXGB obtains the best performance compared to previous models. Our model outperforms all existing models on ACC, SN, SP, MCC, and AUC (5-fold cross-validation: ACC = 0.9903, MCC = 0.9805, Sn = 0.9898, Sp = 0.9907, and auROC = 0.9994), suggesting that the iRice6mA-LMXGB is a powerful and robust predictor that can help researchers to identify and analyze the 6mA locus in the rice genome more effectively, thus providing a deeper understanding of the complex mechanisms of gene regulation and advancing the field of life sciences. It is demonstrated through UMAP visualization that the fine-tuning strategy for large language models significantly enhances the model’s feature extraction ability. This raises the possibility that large language models can be fine-tuned for various purposes and deployed for plant-specific domains to solve biological problems. Moving ahead, we plan to expand our dataset and perform model optimization to enhance the generalizability of our model for broader applications.

## Data Availability

The raw sequence data used in the study were obtained from the following URL: http://lin-group.cn/server/iDNA6mA-Rice.
